# Long-Term Anthropogenic Management and Associated Loss of Plant Diversity Deeply Impact Virome Richness and Composition of *Poaceae* Communities

**DOI:** 10.1128/spectrum.04850-22

**Published:** 2023-03-14

**Authors:** François Maclot, Virginie Debue, Carolyn M. Malmstrom, Denis Filloux, Philippe Roumagnac, Mathilde Eck, Lucie Tamisier, Arnaud G. Blouin, Thierry Candresse, Sébastien Massart

**Affiliations:** a Plant Pathology Laboratory, Terra-Gembloux Agro-Bio Tech, University of Liège, Gembloux, Belgium; b Department of Plant Biology and Program in Ecology, Evolution, & Behavior, Michigan State University, East Lansing, Michigan, USA; c CIRAD, UMR PHIM, Montpellier, France; d PHIM Plant Health Institute, CIRAD, INRAE, Institut Agro, IRD, University of Montpellier, Montpellier, France; e Virology-Phytoplasmology Laboratory, Agroscope, Nyon, Switzerland; f University of Bordeaux, INRAE, UMR BFP, CS20032, Villenave d’Ornon, France; USDA San Joaquin Valley Agricultural Sciences Center

**Keywords:** virus ecology, wild and cultivated *Poaceae*, high-throughput sequencing, virome, virus richness, virus prevalence

## Abstract

Modern agriculture has influenced plant virus emergence through ecosystem simplification, introduction of new host species, and reduction in crop genetic diversity. Therefore, it is crucial to better understand virus distributions across cultivated and uncultivated communities in agro-ecological interfaces, as well as virus exchange among them. Here, we advance fundamental understanding in this area by characterizing the virome of three co-occurring replicated *Poaceae* community types that represent a gradient of grass species richness and management intensity, from highly managed crop monocultures to little-managed, species-rich grasslands. We performed a large-scale study on 950 wild and cultivated *Poaceae* over 2 years, combining untargeted virome analysis down to the virus species level with targeted detection of three plant viruses. Deep sequencing revealed (i) a diversified and largely unknown *Poaceae* virome (at least 51 virus species or taxa), with an abundance of so-called persistent viruses; (ii) an increase of virome richness with grass species richness within the community; (iii) stability of virome richness over time but a large viral intraspecific variability; and (iv) contrasting patterns of virus prevalence, coinfections, and spatial distribution among plant communities and species. Our findings highlight the complex structure of plant virus communities in nature and suggest the influence of anthropogenic management on viral distribution and prevalence.

**IMPORTANCE** Because viruses have been mostly studied in cultivated plants, little is known about virus diversity and ecology in less-managed vegetation or about the influence of human management and agriculture on virome composition. *Poaceae* (grass family)-dominated communities provide invaluable opportunities to examine these ecological issues, as they are distributed worldwide across agro-ecological gradients, are essential for food security and conservation, and can be infected by numerous viruses. Here, we used multiple levels of analysis that considered plant communities, individual plants, virus species, and haplotypes to broaden understanding of the *Poaceae* virome and to evaluate host-parasite richness relationships within agro-ecological landscapes in our study area. We emphasized the influence of grass diversity and land use on the composition of viral communities and their life history strategies, and we demonstrated the complexity of plant-virus interactions in less-managed grass communities, such as the higher virus prevalence and overrepresentation of mixed virus infection compared to theoretical predictions.

## INTRODUCTION

Viruses are among the smallest but potentially most powerful biological entities, and they significantly influence the functioning of plant communities (1). While they are most frequently described as pathogens, viruses may alternatively develop commensal or even mutualistic relationships with hosts (2, 3), with the nature of these relationships dependent on environmental conditions as well as on virus and host genotypes (4, 5). In nature, plant viruses have coevolved over long periods with their vector(s) and host(s) in complex trophic interaction networks (6). However, development of agriculture deeply modified plant communities worldwide, creating agro-ecosystems composed of both cultivated and uncultivated areas. This profound ecosystem change most certainly altered the dynamics of virus-plant-vector interactions and likely fostered virus emergence and even disease epidemics (7, 8). To develop better mechanistic understanding of these processes requires fundamental knowledge of virus communities and their responses to host and management shifts that result from increased agricultural intensity. At present, virus dynamics are better understood within crop systems than in less-managed systems, and characterization of viruses infecting wild plants is only just beginning. However, evidence indicates that virus infections are common in nature and frequently asymptomatic, often involving multiple virus species (mixed infections) (9–11). Moreover, there is developing understanding that host diversity can powerfully influence pathogen prevalence or diversity, either increasing (amplification effect) or decreasing (dilution effect) infectious disease risk (12).

Here, we advance fundamental understanding of plant virus dynamics by characterizing the virome of three co-occurring *Poaceae* (grass family) community types that represent a gradient of grass species richness and management intensity, from highly managed crop monocultures to little-managed, species-rich grasslands. The *Poaceae* virome is one of the most important to understand globally, because collectively, humanity depends on *Poaceae* species, directly or indirectly, for two-thirds of its caloric consumption, making this plant family essential for world food security (13, 14). Cereal crops are susceptible to at least 74 virus species worldwide (15), with yield losses reaching up to 80% (16). Crop-infecting viruses may further spill into wild (noncultivated) grasses and reduce wild host fitness, thus posing conservation risks (17–19). Alternatively, wild or weedy grasses might serve as a reservoir for crop viruses between cropping cycles. In contrast with crop viruses, only a few studies have begun to evaluate the nature and ecology of non-crop-infecting (“wild”) viruses harbored by grasses (20, 21).

High-throughput sequencing (HTS) is well-suited for characterizing plant viruses, both known and unknown (22). Whether plants are individually sampled and barcoded (ecogenomics [23]), pooled (24), or considered within spatially explicit contexts (geometagenomics [20]), viral metagenomics provides key information that can illuminate how anthropogenic perturbations impact plant-virus interactions and emergence of viral diseases (25). Here, we used HTS to survey, without *a priori* information, all of the viruses inhabiting the target grass communities, i.e., to characterize their viral metagenomes or viromes (26). Our first objective was to characterize the *Poaceae* viromes of our study sites, including both known and novel RNA and DNA viruses. In this effort, we strove to determine viral taxa to the finest level (i.e., species) where possible. Second, we aimed to evaluate the relationship between host plant richness and virus richness, as evident among these plant communities, and its temporal stability over 2 years. Third, we investigated whether the species composition of the plant community viromes was more strongly associated with plant community type (crop, pasture, grassland) or site location. Finally, we examined three individual virus species in detail to determine their prevalence and spatial distributions within communities, and we investigated the genetic diversity of haplotypes of one latent virus within and between communities. We expected to find high virus prevalence and more severe symptoms in crop fields and lower virus prevalence with fewer symptoms and high rates of coinfection in the less-managed communities. Combining different analysis levels (plant communities, individual plants, virus species, and even haplotypes), we presented a broader view of plant-virus interactions at agro-ecological landscapes. This is of particular interest as most host-parasite richness relationships have so far been considered for a limited number of plant viruses and hosts (27, 28). In addition, viral metagenomics studies have limited the virus diversity examination to virus family level (20) or used theoretical operational taxonomic units (OTUs) as an acceptable proxy to viral species (29–31).

## RESULTS

### A diversified and largely unknown virome in *Poaceae*.

**FIG 1:**
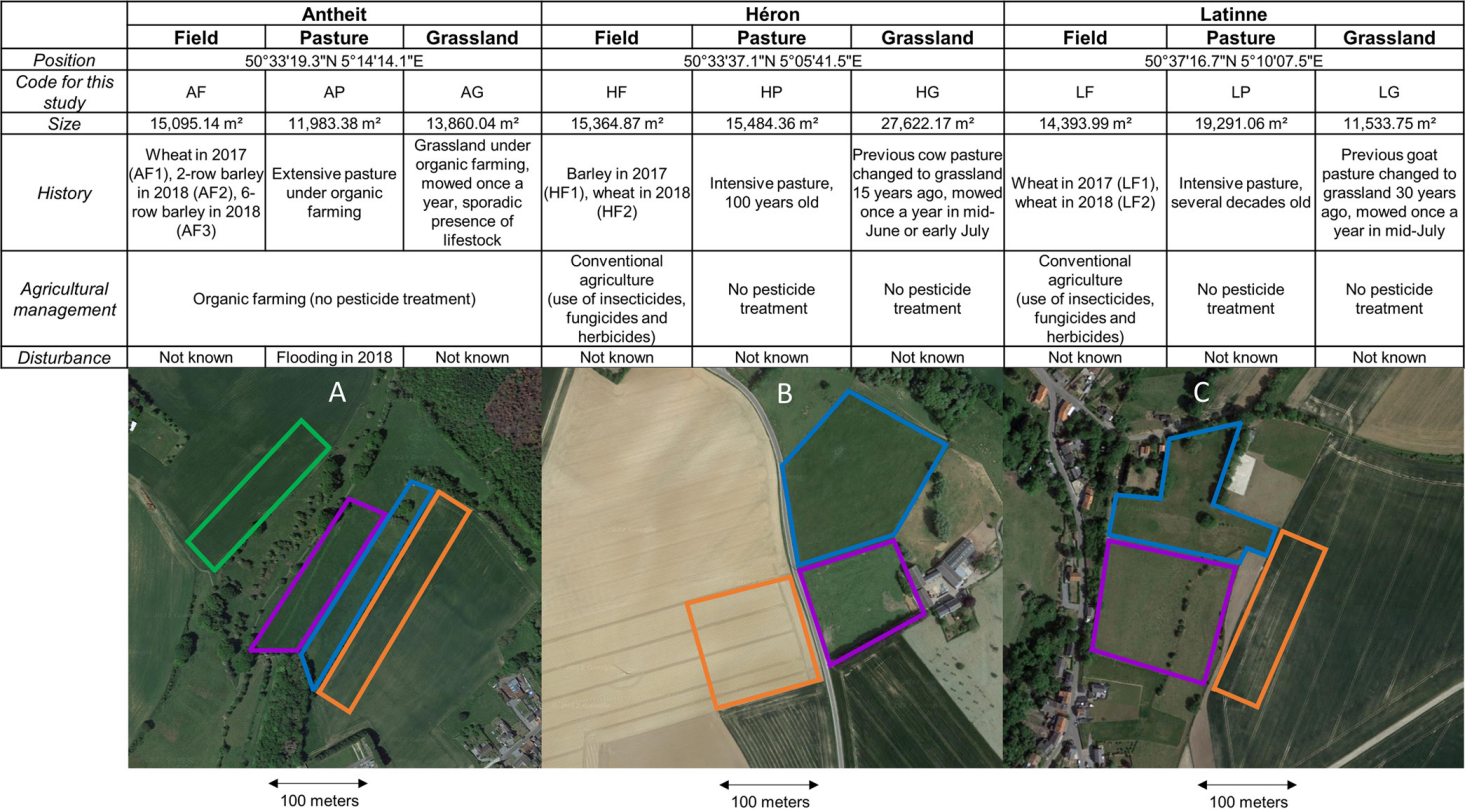
Information table (GPS position, code, size, history, agricultural management, and eventual disturbance) in the different communities and locations studied. Below the table is a representation of Antheit (A), Héron (B), and Latinne (C) locations, with the three adjacent ecosystems examined: a barley or wheat field (in orange), an intensive pasture (in purple), and a grassland with high biological value (in blue). An additional 6-row barley field (in green) was sampled in 2018 in Antheit.

We characterized the virome of three co-occurring *Poaceae*-dominated communities that represented a gradient of plant species richness and management intensity (cereal crops, grazed pastures, and mowed grasslands). We studied these community types across 2 years (2017 and 2018) at three replicated sites 10 km apart (Antheit, Héron, Latinne) within the Belgian Natural Park Burdinale-Mehaigne (Fig. 1). To assess the virome in each community, we sampled 950 plants belonging to 24 *Poaceae* species (see Table S1 in the supplemental material) and used a viral metagenomic approach (virion-associated nucleic acids [VANA]) on pools of 50 samples per community (Table S2). Libraries contained on average 17.9% of viral reads (ranging from 0% to 78% [Table S3]). A low level of cross-sample contamination was observed, as the alien target sequences (PLRV, BSV) detected in the *Poaceae* samples represented on average 0.01% of the total reads in the libraries. This number was used as the detection threshold for the present study, meaning that no viral detection below this level was considered. Fifty-one consensus plant virus genomes were assembled (1,496 to 13,876 nucleotides [nt] in length), covering for 47 of all known open reading frames (ORFs) described in the corresponding genera or families, thus permitting their assignment to species. We also identified four incomplete virus genomes (1,497 nt to 6,785 nt in length) that represented putative novel species belonging to *Closterovirus*, *Rymovirus*, *Varicosavirus*, and *Amalgavirus* genera (based on International Committee on Taxonomy of Viruses [ICTV] demarcation criteria on RNA-dependent RNA polymerase [RdRp] and coat protein genes and host range). These 51 RNA viruses were assigned to 16 virus families representing 21 virus genera (Table 1). These results demonstrated that the *Poaceae* virome remains largely unknown, with more than two-thirds of the detected viruses (*n* = 37) corresponding to putative novel virus species (Table 1 and Table S4). These novel virus species were primarily persistent viruses and mycoviruses (i.e., 3 novel alphachrysoviruses, 10 novel partitiviruses, and 13 novel totiviruses), which were found in all three community types. Virome comparison across time revealed that 77% of the virus species were detected in both sampling years (74% for the plant viruses and 79% for the mycoviruses). This number increased to 85% when only long-term plant communities (pastures and grasslands) were considered.

**TABLE 1:** The different virus families, genera, and species detected in the *Poaceae* communities (cereal fields, grazed pastures, and mowed grasslands)

Virus family	Virus genus	Virus species	Known or novel species	Category^*a*^	Plant or mycovirus	Communities where present
*Alphaflexiviridae*	*Lolavirus*	*Lolium latent virus*				Pastures, grasslands
	*Potexvirus*	*White clover mosaic virus*				Pastures
*Bromoviridae*	*Bromovirus*	*Brome mosaic virus*				Wheat fields
*Potyviridae*	*Rymovirus*	*Ryegrass mosaic virus*				Pastures, grasslands
*Solemoviridae*	*Polerovirus*	*Cereal yellow dwarf virus* RPS				Pastures, grasslands
		*Cereal yellow dwarf virus* RPV		+ssRNA		Pastures, grasslands
		*Wheat yellow dwarf virus*				Grasslands
*Tombusviridae*	*Luteovirus* ^*b*^	*Barley yellow dwarf virus* GAV-MAV	Known		Plant virus	Pastures
		*Barley yellow dwarf virus* Ker II-Ker III				Pastures, grasslands
		*Barley yellow dwarf virus* PAS-PAV				Pastures, grasslands
	*Panicovirus*	*Cocksfoot mild mosaic virus*				Grasslands
*Endornaviridae*	*Alphaendornavirus*	*Hordeum vulgare endornavirus*				6-row barley
*Reoviridae*	*Fijivirus*	*Oat sterile dwarf virus*		dsRNA		Pastures, grasslands
*Partitiviridae*	Unclassified	*Black grass cryptic virus 1*				Pastures, grasslands
*Closteroviridae*	*Closterovirus*	*Poaceae Liege closterovirus*				Pastures, grasslands
*Potyviridae*	*Rymovirus*	*Poaceae Liege rymovirus*				Pastures, grasslands
*Secoviridae*	*Nepovirus*	*Poaceae Liege nepovirus A*				Pastures, grasslands
	Unclassified	*Poaceae Liege virus 1*				Pastures, grasslands
*Solemoviridae*	*Sobemovirus*	*Poaceae Liege sobemovirus*		+ssRNA	Plant virus	Grasslands
*Tombusviridae*	*Machlomovirus*	*Poaceae Liege machlomovirus*	Novel			Pastures, grasslands
	*Umbravirus*	*Poaceae Liege umbravirus 1*				Grasslands
		*Poaceae Liege umbravirus 2*				Pastures
*Tymoviridae*	*Tymovirus*	*Poaceae Liege tymovirus*				Pastures
*Rhabdoviridae*	*Varicosavirus*	*Poaceae Liege varicosavirus*		−ssRNA		Pastures, grasslands
*Amalgaviridae*	*Amalgavirus*	*Poaceae Liege amalgavirus 1*			Plant virus	Grasslands
	*Deltapartitivirus*	*Poaceae Liege partitivirus 15*				Pastures, grasslands
		*Poaceae Liege partitivirus 1*				Pastures, grasslands
		*Poaceae Liege partitivirus 3*				Pastures, grasslands
		*Poaceae Liege partitivirus 4*				Grasslands
		*Poaceae Liege partitivirus 5*				Grasslands
*Partitiviridae*	Unclassified	*Poaceae Liege partitivirus 6*			Plant virus/Mycovirus	Grasslands
		*Poaceae Liege partitivirus 7*				Grasslands
		*Poaceae Liege partitivirus 9*				Pastures, grasslands
		*Poaceae Liege partitivirus 11*				Grasslands
		*Poaceae Liege partitivirus 14*				Grasslands
	*Betapartitivirus*	*Poaceae Liege partitivirus 13*				Pastures, grasslands
		*Poaceae Liege alphachrysovirus 1*				Grasslands
*Chrysoviridae*	*Alphachrysovirus*	*Poaceae Liege alphachrysovirus 2*	Novel	dsRNA		Pastures, grasslands
		*Poaceae Liege alphachrysovirus 3*				Grasslands
		*Poaceae Liege totivirus 2*				Grasslands
		*Poaceae Liege totivirus 3*				Grasslands
		*Poaceae Liege totivirus 4*				Grasslands
		*Poaceae Liege totivirus 5*				Grasslands
		*Poaceae Liege totivirus 6*			Mycovirus	6-row barley
		*Poaceae Liege totivirus 12*				Pastures, grasslands
*Totiviridae*	*Totivirus*	*Poaceae Liege totivirus 13*				Pastures, grasslands
		*Poaceae Liege totivirus 14*				Pastures, grasslands
		*Poaceae Liege totivirus 15*				6-row barley, grasslands
		*Poaceae Liege totivirus 16*				6-row barley, grasslands
		*Poaceae Liege totivirus 17*				6-row barley, grasslands
		*Poaceae Liege totivirus 18*				6-row barley, grasslands
		*Poaceae Liege totivirus 19*				Grasslands

a +ssRNA, positive-sense single-stranded RNA; −ssRNA, negative-sense single-stranded RNA; dsRNA, double-stranded RNA.

b For the BYDV species complex, recombination events did not allow us to analyze data to the species level, and in some cases the species were thus regrouped as GAV-MAV, KerII-KerIII, and PAS-PAV.

### Relationship between *Poaceae* communities and virome richness or composition.

**FIG 2:**
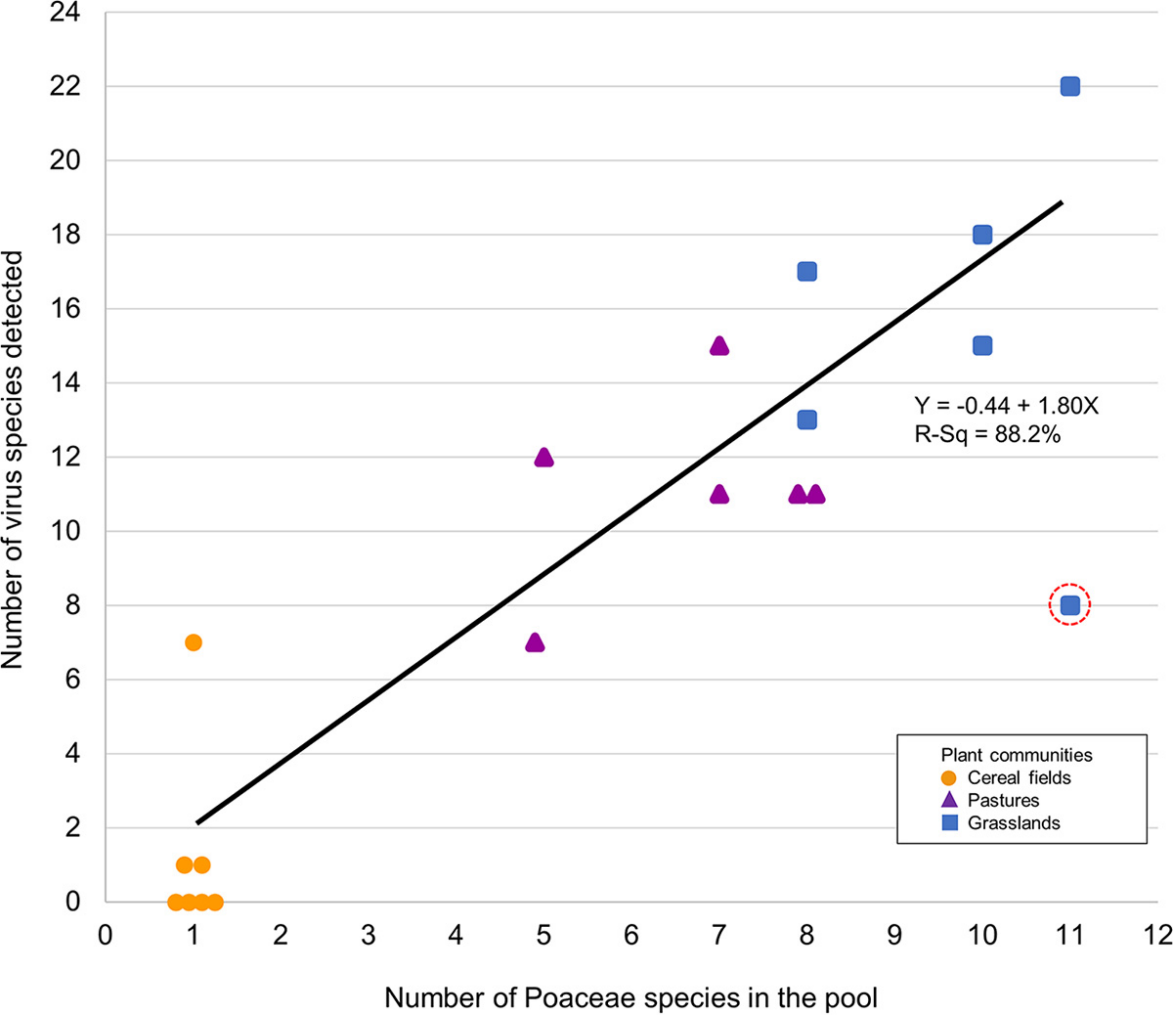
Relationship between *Poaceae* species richness and virus species richness among plant communities for the 2 years studied (cereal fields in orange circle, pastures in purple triangle, grasslands in blue square). The survey area Héron Grassland in 2017 with a different mowing management is highlighted with a dotted red circle. The exclusion of Héron Grassland in 2017 further improved the *R*^2^ value, from 76.0% to 88.2%.

Preliminary HTS results revealed contrasting virome richness levels among plant communities. Significant differences in virus taxa richness were observed among cereal fields, pastures, and grasslands (*P* < 0.001 at virus family, genus, and species levels, Kruskall-Wallis test). Very few or no virus species were found in cereal fields, while a diversified virome was observed in more-diverse communities, with up to 22 virus species detected in grasslands (Fig. 2). The replicate of Héron grassland in 2017 (code HG1) had a different mowing management and presented the lowest grassland virome (8 virus species). Interestingly, pastures and grasslands did not significantly differ in virome richness at family and genus levels (*P* = 0.456 and 0.419, Mann-Whitney test). However, at the species level, grasslands were marginally more diverse than pastures (Mann-Whitney test, *P* = 0.078, but decreased to *P* = 0.010 when we excluded HG1). Linear regression models were used to show the relationship between plant species richness and virus family richness (virus families = 0.60 + 0.62 grass species, *R*^2^ = 74.3%, *P* = 0.000), virus genera richness (virus genera = 0.55 + 0.82 grass species, *R*^2^ = 72.9%, *P* = 0.000), and virus species richness (virus species = 0.22 + 1.57 grass species, *R*^2^ = 76.0%, *P* = 0.000) (Fig. 2).

**FIG 3:**
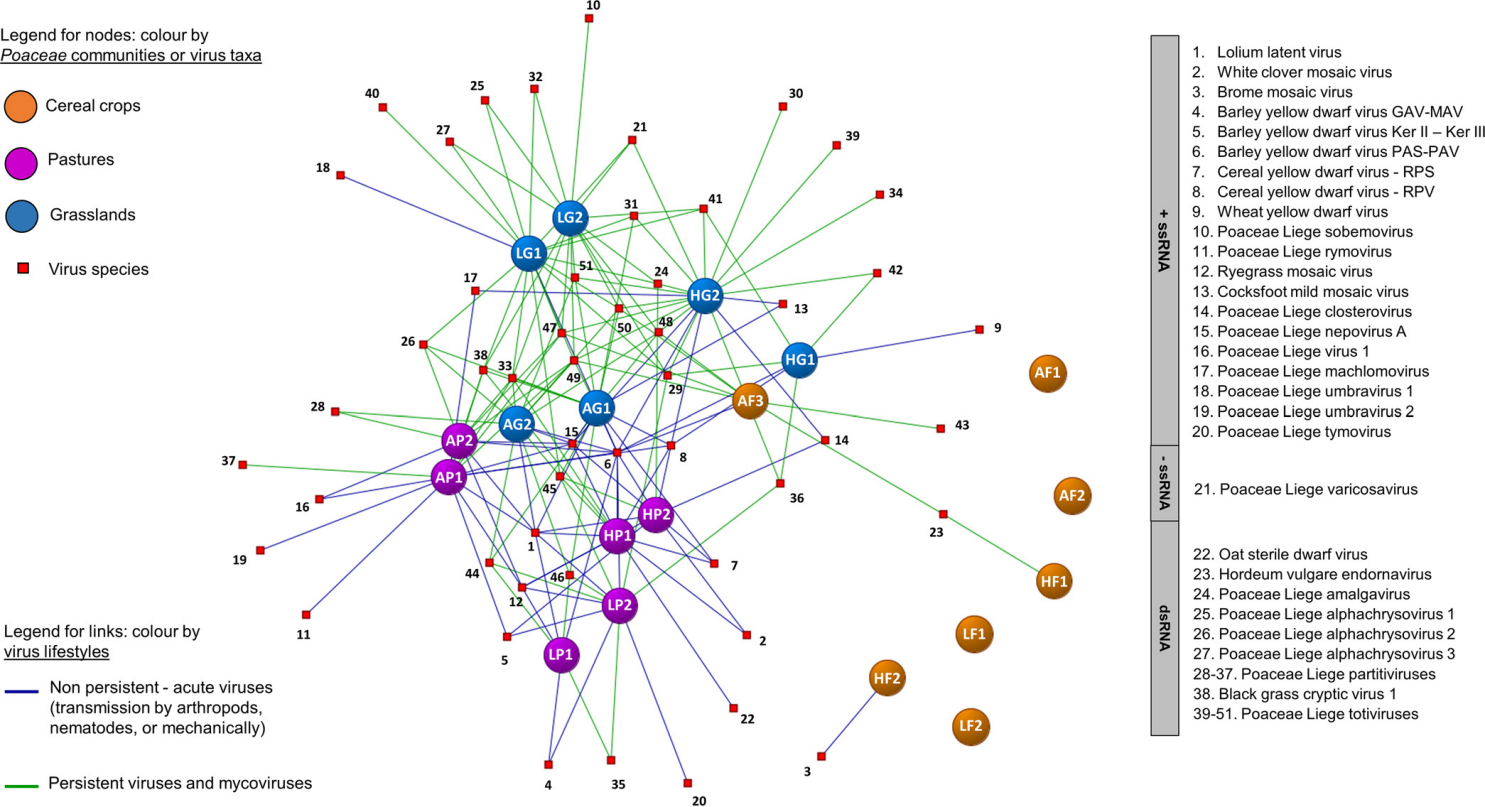
Network analysis of the virus species identified in *Poaceae* communities (cereal crops, pastures, and grasslands) across the three sites and the 2 years studied. Nodes correspond to virus species (red square) or plant community (colored balls). Links are colored according to the putative lifestyle for each virus species (nonpersistent viruses, persistent viruses, and mycoviruses).

**FIG 4:**
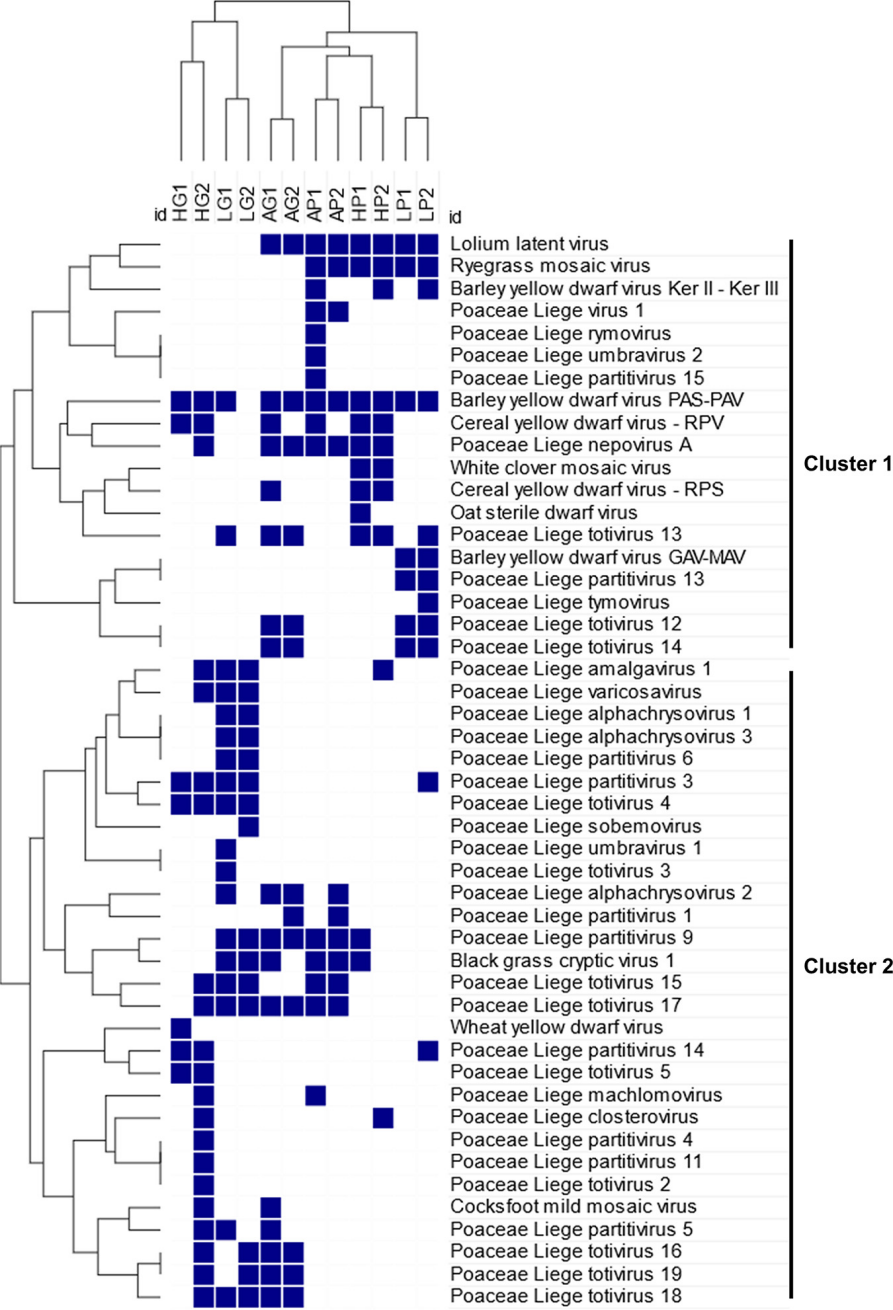
Hierarchical clustering analysis in both plant and virus dimensions. Columns refer to the two different wild *Poaceae* communities (pastures [P] and grasslands [G]) examined among sites (Antheit [A], Héron [H], and Latinne [L]) for the 2 years (2017 [1] and 2018 [2]). Rows correspond to the different virus species detected. Two main clusters of co-occurent virus species are highlighted on the right of the dendrogram.

Virome compositions among *Poaceae* communities were visualized using a network analysis of the virus species identified within each grass community (Fig. 3), and we performed a clustering analysis in both virus and plant dimensions for the grasslands and pastures to highlight any community clustering or virus co-occurrence patterns (Fig. 4). Exclusion of cereal fields improved branch length in the clustering model (see Fig. S1 for the three communities clustering). When considering *Poaceae* communities, replicates belonging to the same community but from different sites and years clustered together (Fig. 3 and 4). We observed two main clusters for the less-managed communities: the first cluster with the grasslands from Héron and Latinne, and the second cluster with the pastures and Antheit grassland (Fig. 4). The 6-row barley fields clustered with Héron and Latinne grasslands, and the wheat field in Héron formed a single cluster (Fig. S1). Considering virus species, network analysis highlighted a series of ubiquitous virus taxa detected in several or even all *Poaceae* community types, such as the persistent viruses and mycoviruses (partitiviruses, totiviruses, alphachrysoviruses) and some nonpersistent (or acute) viruses (*Poaceae* Liege nepovirus A, Lolium latent virus, yellow dwarf viruses). In addition to this shared virome, virus species (mostly nonpersistent viruses) were detected in specific communities, for example, brome mosaic virus in wheat crop, ryegrass mosaic virus in pastures, and cocksfoot mottle virus in grasslands. Interestingly, the network analysis showed association between *Poaceae* communities and virus biology. Pasture viromes were dominated by nonpersistent viruses (i.e., 66% of the nonpersistent virus edges in pastures), while grasslands were more associated with persistent viruses and mycoviruses (i.e., 73% of the persistent virus edges in grasslands) (Fig. 3). This pattern was confirmed by the clustering analysis that identified two main clusters of co-occurring viruses mainly associated with each *Poaceae* community (Fig. 4).

### Higher viral prevalence and multiple infection in less-managed plant communities.

**FIG 5:**
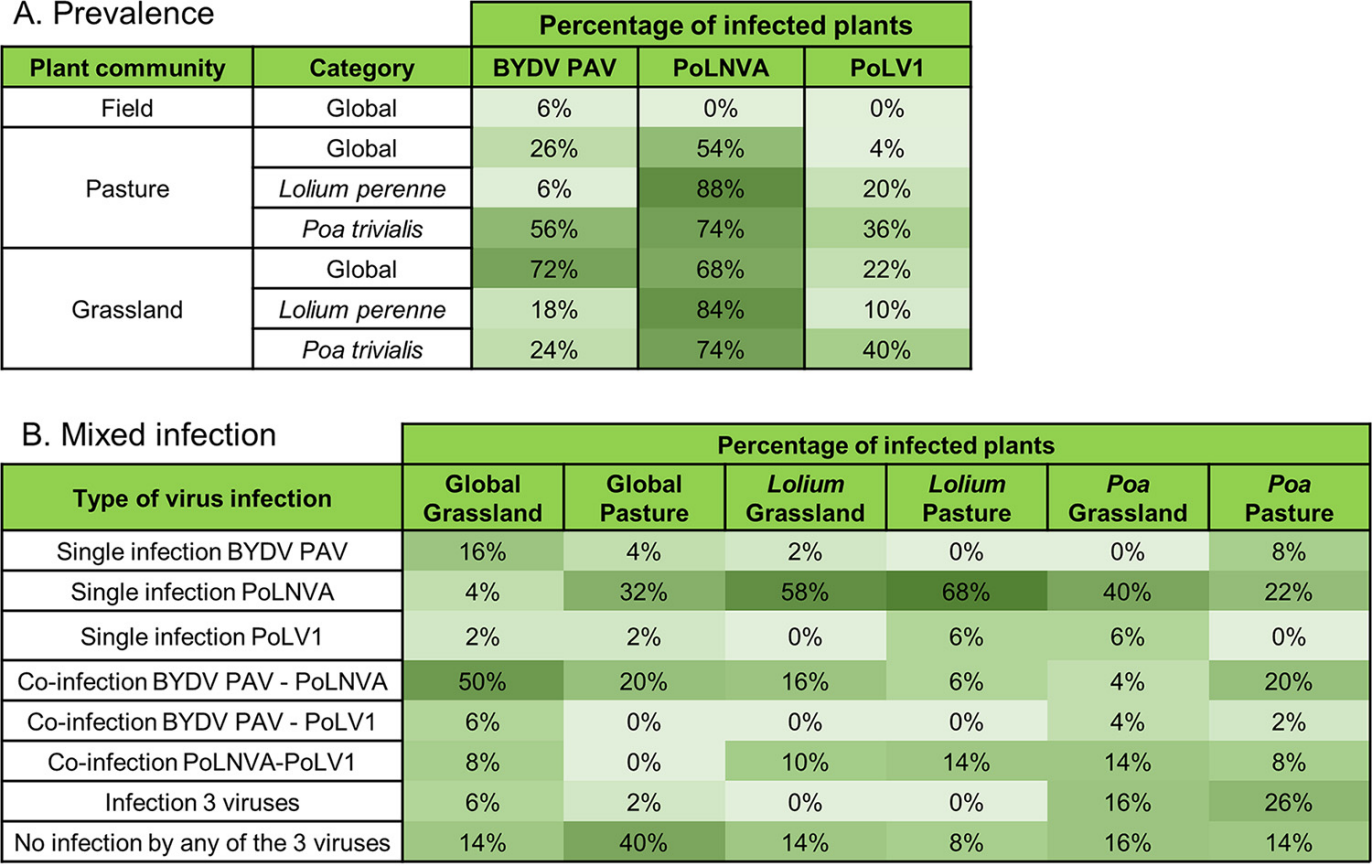
(A) Virus prevalence of BYDV-PAV and the two novel species (PoLNVA and PoLV1) observed among plant communities and species at the Antheit sites in 2018. Plant community pools were designated “Global.” Plant species pools were also examined for Lolium perenne and *Poa trivialis*. (B) Percentages of plants in single and multiple infections with BYDV-PAV, PoLNVA, and PoLV1 in uncultivated plant communities (grasslands and pastures, named global) and two wild *Poaceae* species (Lolium perenne and *Poa tivialis*), in Antheit in 2018.

To complete the first ecological information provided by the *Poaceae* virome composition in plant pools, we analyzed virus prevalence, coinfection, and distribution within the plots. For that, we performed virus detection in individual plants from the Antheit site in 2018 for three viruses detected by HTS: barley yellow dwarf virus-PAV (BYDV-PAV, found in all communities) and two novel secovirids (*Poaceae* Liege nepovirus A [PoLNVA] and *Poaceae* Liege virus 1 [PoLV1]), which were recently characterized (32) and were detected in both pastures and grasslands. Individual plants from community pools (grassland, pasture, and 6-row barley field) and from two dominant species (Lolium perenne, Poa trivialis) were analyzed. Infirming our hypotheses, we observed high virus prevalence with an absence of visible symptoms in less-managed communities (i.e., pastures and grasslands). The PoLNVA nepovirus was highly prevalent in both pastures and grasslands and very frequently detected within the host species *L. perenne* and *P. trivialis* (maximum of 88% found in *L. perenne* in pasture). In contrast, BYDV-PAV and PoLV1 had divergent prevalence patterns across plant community types and species (Fig. 5A). In barley, the hypothesis of high prevalence was not fulfilled, with a prevalence of only 6% observed for BYDV-PAV. The hypothesis of high coinfection rates in the wild compartment was confirmed, in particular for coinfection by BYDV-PAV and PoLNVA (up to 50% of plants coinfected in the grassland pool). Interestingly, multiple infections by the three viruses were relatively rare (0 to 7%), except for *P. trivialis*, for which with 16% of the grassland individuals and 26% of the pasture individuals were multiply infected (Fig. 5B). These values were somewhat higher than the predicted coinfection rate (i.e., the product of individual rates for the three viruses, which gave predictions of 7% for grassland (0.24 × 0.74 × 0.4) and 15% for pasture (0.56 × 0.74 × 0.36). For single infections, low rates were observed for BYDV-PAV and PoLV1 (0 to 16%), but single infection by PoLNVA was more prevalent and reached 68% in *L. perenne* in pasture (Fig. 5B).

**FIG 6:**
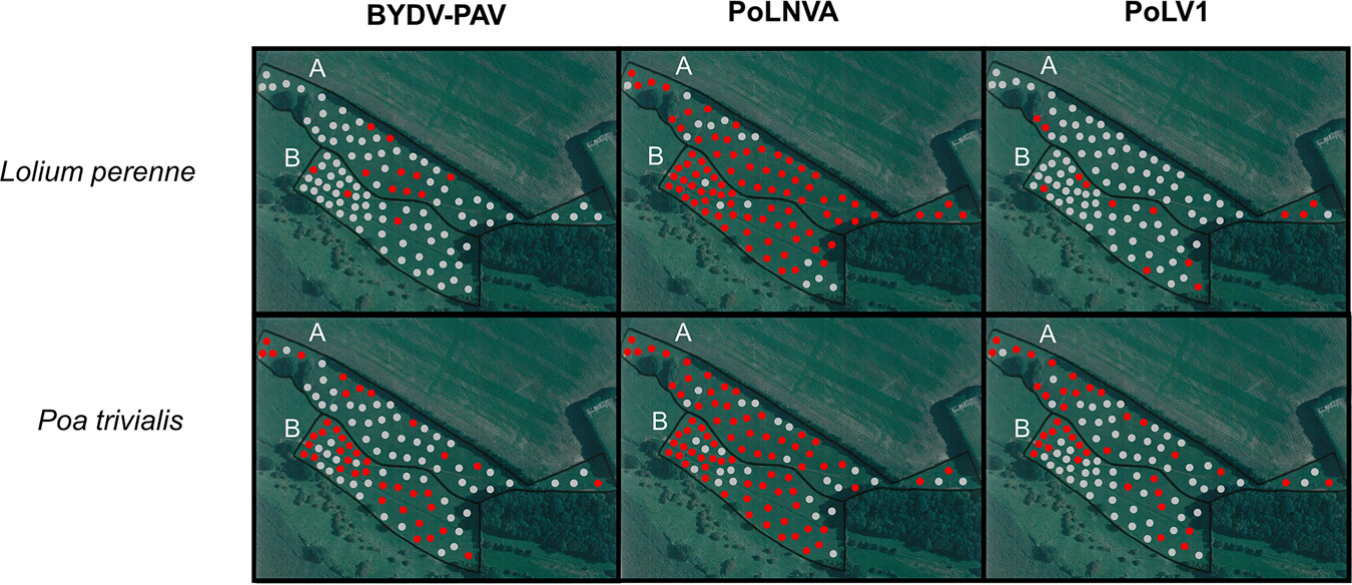
Geographical distribution of infected plants (for BYDV-PAV, PoLNVA, and PoLV1) belonging to Lolium perenne and *Poa trivialis* in Antheit grassland (A) and pasture (B). Gray and red dots correspond to noninfected and infected plants, respectively.

Virus spatial patterns were analyzed for *P. trivialis* and *L. perenne* in grassland and pasture. The nepovirus (putatively transmitted by seeds, pollen, and/or nematodes) was distributed throughout the plots, with just a few clusters of noninfected plants (index of aggregation [*I_a_*] =1.72 to 1.90; *P* value for aggregation under the null hypothesis [*P_a_*] = 0.02 or 0.03), except for the *P. trivialis* pasture with a random distribution of noninfected spots (*I_a_* = 1.00; *P_a_* = 0.38). In contrast, virus infections transmitted by insects (i.e., aphids for BYDV-PAV or putatively aphids or leafhoppers for PoLV1) were more clustered (Fig. 6). Spatial analysis by distance indices (SADIE) analyses revealed virus aggregation within the plots, in particular for PoLV1 in grasslands for both *P. trivialis* and *L. perenne* (*I_a_* = 1.842 and 1.839, respectively; *P_a_*= 0.0171). Virus distributions were also compared to each other, and contrasted associations were shown between insect-transmitted viruses according to the plant species: mostly positive association for *P. trivialis* (highest positive value of 0.54 with *P* = 0.00 for BYDV-PAV and PoLV1 in pasture) and negative association for *L. perenne* (highest negative value of −0.62 with *P* = 0.99 for BYDV-PAV and PoLV1 in grassland) (Table S5).

### Large viral genetic structure in wild grasses.

**FIG 7:**
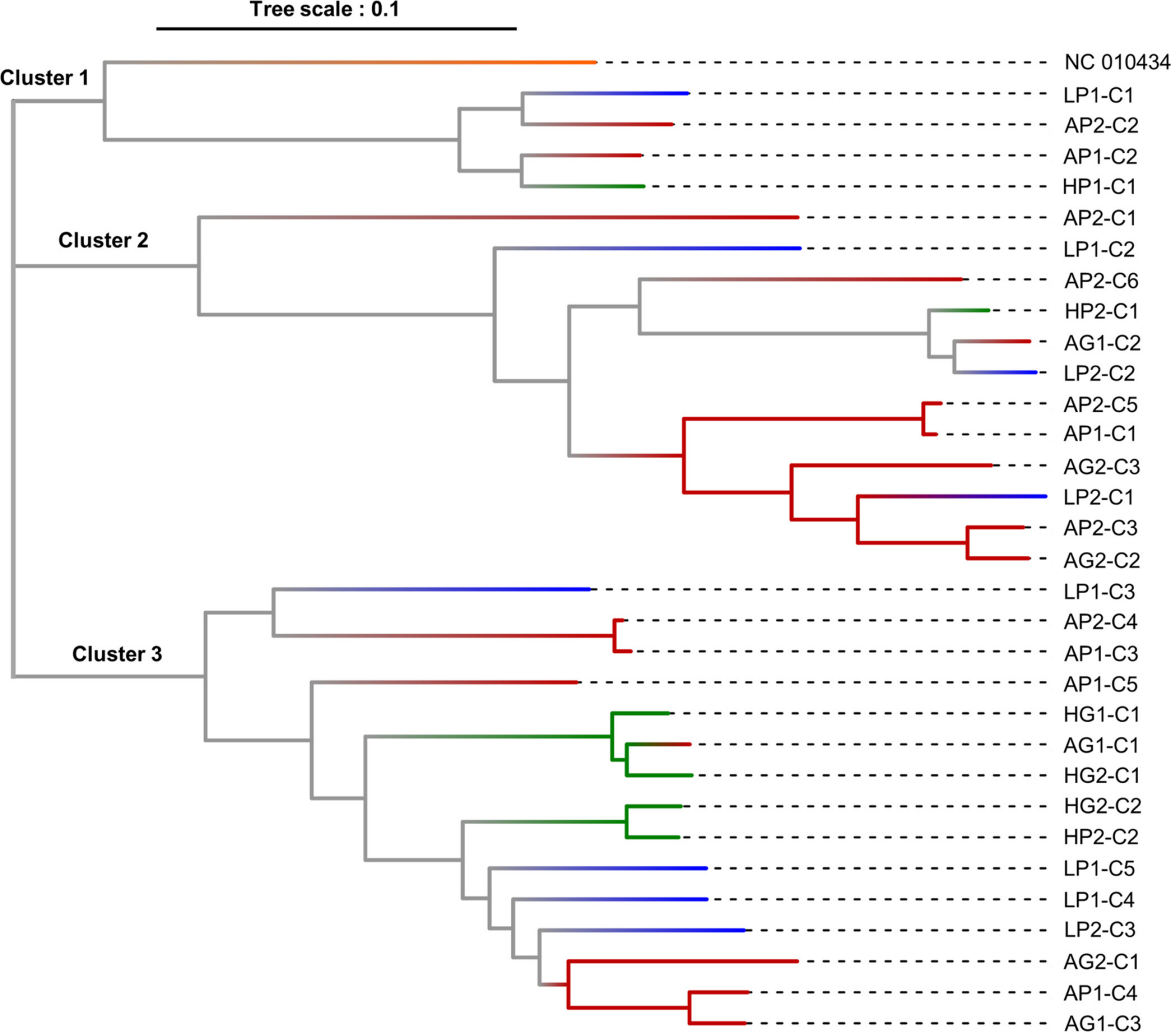
Phylogenetic tree (maximum-likelihood method, Tamura-Nei model, 1,000 bootstraps, threshold of 70% occurrence) of the RdRp region of Lolium latent virus in *L. perenne* in different sites (Antheit [A] in red, Héron [H] in green, and Latinne [L] in blue) and communities (grassland [G] and pasture [P]) for the 2 years (2017 [1] and 2018 [2]) on consensus sequences (contigs *de novo* assembled [*Cx*]). Samples were compared to the NCBI reference of LLV (isolate US1, NC_010434), shown in orange.

Interannual comparison of viral sequences within the same plant communities revealed a high genome-level genetic stability for numerous virus species. For instance, 99.4% and 99.8% nucleotide identities were observed for ryegrass mosaic virus (RMV) and for PLNA, respectively, in Antheit pasture between both sampling years. However, more variability was found for Lolium latent virus (LLV; genus *Lolavirus*, family *Alphaflexiviridae*), which made it interesting to examine the virus genetic structure among communities and sites. This analysis was performed in *L. perenne*, as this species seemed to be the major host for LLV (higher prevalence and more complete genome obtained compared to other grass species). Preliminary results of BLASTn searches revealed a high level of variability of LLV contigs that shared 70% to 99% nucleotide identity with LLV isolates in the NCBI GenBank database. Calculating the pairwise identity shared by the RdRp region of LLV isolates detected in this study further confirmed their genetic diversity. Hence, the LLV RdRp pairwise identity matrix (Table S6) presented a series of clusters and subclusters with 75 to 99% nucleotide identity between LLV RdRp sequences recovered from this study. Phylogenetic analyses performed on LLV RdRp sequences (Fig. 7) showed three main clusters of LLV. Their composition showed various interesting features with closely related RdRp sequences detected: (i) in the same plot for the 2 years studied (e.g., AP1-C1 and AP2-C5, HG1-C1 and HG2-C1); (ii) in pasture and grassland from the same location (e.g., AG1-C3 and AP1-C4, HP2-C2 and HG2-C2); (iii) in different sites (e.g., AG1-C2, HP2-C1, and LP2-C2); or (iv) detected only once for a given plot and/or location (e.g., HP1-C1 or LP1-C1). In addition, very different RdRp sequences were observed at the same time in the same plot (e.g., AG1-C1, AG1-C2, and AG1-C3), some of which were closely related to sequences identified in other sites (e.g., AG1-C2, HP2-C1, and LP2-C2).

## DISCUSSION

Our findings expand the understanding of the *Poaceae* virome in agro-ecological landscapes and relationships between host species richness and parasite species richness in our study systems. At the plant community level, we discovered a diverse and temporally stable virome dominated by largely unknown species, with more than 51 novel virus species identified, many of which were persistent viruses and mycoviruses. Community-level grass species richness was associated with both virome species richness and composition, revealing a complex virome network in *Poaceae*. At the plant level, we found that virus prevalence, coinfections, and spatial distributions varied with grass community and host species, sometimes in ways that differed from theoretical predictions in virus ecology.

So far, plant virus metagenomics-based studies have focused their ecological analyses on family or genus taxonomic ranks, or examined viral OTUs clustered at a standard level of 90% identity (20, 29, 33, 34). Improving the taxonomic resolution of such studies toward the species level is a crucial point to fine-tune ecological analyses, such as the estimation of virus(es) genetic structure and diversity (richness and evenness), or even to characterize novel virus species (22). In this study, we strove to obtain as complete virus genomes as possible, covering for many of them all known ORFs described in the corresponding genera or families. The virome could thus be analyzed at species level, allowing more in-depth ecological analyses (correlation between plant and virus species richness, virus network, phylogeny, etc.). One limitation was for segmented viruses (e.g., partitiviruses). It was not possible to associate the different segments in sample pools containing several partitiviruses, and we thus focused the analysis on segment 1 (encoding the RdRp which corresponded to a hallmark gene for the *Riboviria* realm) (35). Note that hundreds of small viral contigs (i.e., 300 to 500 nt) presenting homologies to members of *Chrysoviridae*, *Endornaviridae*, *Partitiviridae*, *Rhabdoviridae*, and *Totiviridae* families and to unclassified mycoviruses were also detected in wild *Poaceae*, but they could not be classified at the species level. These contigs suggested the presence of additional virus species at low abundance or low titer within the communities.

A diversified virome was thus identified in the Belgian *Poaceae* communities, with at least 51 viruses belonging to 21 genera and 16 families (Table 1). As the VANA approach can potentially detect both RNA and DNA viruses, the virome of temperate *Poaceae* species appeared in our study to be vastly dominated by RNA viruses. In addition, this *Poaceae* virome was found to be more than two-thirds composed of unknown species, in particular persistent and fungal-associated virus taxa from *Chrysoviridae*, *Partitiviridae*, and *Totiviridae* families. These genera were found in all less-managed communities and in one cereal field, highlighting their broad distribution in plants and confirming findings from other recent virome-based studies (20, 29, 34, 36).

In this study, three *Poaceae*-based communities with differing plant species richness levels due to anthropogenic management were compared and revealed that virome richness increased with the number of grass species within the sampled community. The positive correlation observed between plant and virus species richness agreed with findings from other studies analyzing host and parasite richness in plants and animals (37–39). Our findings with viruses are important, because most disease ecology studies so far that have considered host-pathogen richness relationships have focused on foliar fungal pathogens or on a more targeted set of plant viruses (e.g., 5 viruses [27], 11 viruses [28]) or analyzed viral OTUs as proxy to viral species (31). A recent geometagenomics study performed in France and South Africa highlighted that some cultivated areas (with lower host diversity) could present a greater virus family diversity than uncultivated ones (20). In the present study, comparison of the virus family or genus diversity between communities showed less clear tendencies than using virus species, as illustrated in grasslands versus pastures, making the results difficult to compare with those reported in reference 20.

Network and clustering analyses also highlighted different virus lifestyles among the wild *Poaceae* communities. More acute or nonpersistent virus taxa were identified in pastures and in Antheit grassland, while Héron and Latinne grasslands harbored more persistent viruses and mycoviruses (Fig. 3 and 4). Plant diversity and land use can thus influence both the virome richness and composition, as illustrated here by the mowing management. In Héron grassland, mowing date variation changed the virus richness and composition observed in 2017 and 2018, and in Antheit the sporadic presence of livestock in the grassland led to a virus composition closer to that of pastures. Bernardo et al. (20) reported that some virus families were significantly associated with cultivated or with unmanaged areas in France and South Africa. The spatial distribution of host populations is expected to be a key determinant of disease dynamics, with infection risk tending to increase with increasing host population size and connectivity to other populations (40). Infection spread of vector-transmitted viruses might be favored in dense networks of host populations (41), which can explain why we observed more nonpersistent viruses in pastures. In contrast, more diverse, long-term, and less-connected plant populations, as found in grasslands, could lead to increased distribution of persistent viruses that are transmitted vertically via gametes. In cereal fields, the high host density could lead to high virus spread, but it may be counterbalanced using pesticides and the yearly sowing of new certified seeds. In particular, the impact of insecticides on the vectors of phytoviruses, of fungicides on the spatial prevalence of fungi, and therefore of mycoviruses, are expected to affect the virome, as suggested by the results reported in reference 36. This could explain the differences in the virome observed between the organic (in Antheit) and conventional (in Héron and Latinne) cereal fields.

Temporal dynamics in the *Poaceae* virome were also examined and revealed relative stability with 85% of virus species detected in both years in the permanent plant communities, i.e., grazed pastures and mowed grasslands. This confirmed the absence of significant differences in plant virus population composition for a few targeted viruses in natural plant communities (34). Identification of some plant viruses to species level also allowed the analysis of intraspecific variability, as explored here with LLV. Several situations were observed, with different virus genomes coexisting in the same studied plant communities, with some of them detected both years while others were identified in a single year only, as well as one situation of closely related genomes detected in different sites (Fig. 7). Results suggest potential circulation of virus isolates among communities within the same site, but not frequent exchange among sites. In addition, 18/31 of the LLV variants were not found from 1 year to another, indicating that the variant diversity was incompletely sampled and was likely higher than reported. This observation is relevant because it shows that the sampling strategy was well adapted for studying the composition at species level but that studying the population genetic structure of a virus would require an additional sampling effort.

Analysis of individual plants found differing prevalence rates, coinfections, and spatial distributions among plant communities, plant species, and virus species. Nematode-transmitted and single-stranded RNA seed- or pollen-transmitted viruses, such as nepoviruses, have been demonstrated to have large host range breadth (42), in line with the observation that numerous *Poaceae* species were found infected by the novel nepovirus (32). Moreover, the two other viruses studied, BYDV-PAV and PoLV1, presented more contrasting prevalence rates, depending on plant community or species (Fig. 5). Examination of transmission modes can, at least partially, contribute to explain these differences. Unlike nepoviruses, BYDV-PAV is transmitted by aphids (and novel PoLV1 is close to waikaviruses, which are also insect-vectored). Complex interactions are involved in the insect transmission of plant viruses, in particular vector feeding behavior and attraction or deterrence of vectors for host plants (43). These effects are likely even more complex in natural compartments composed of multiple plant and insect species.

In this study, the plant species richness of grasslands was greater than that of pastures, and the grassland communities included some potential insect hosts not found in pastures. Along with other factors, such as vector feeding preferences, increased dicot diversity in grasslands, and different mowing management, this could have contributed to greater virus prevalence in grassland than pasture. Interspecific interactions among insect vectors (competition, coexistence, etc.) may have also impacted virus distribution within the plot (Fig. 6), as illustrated by SADIE analyses (e.g., negative association between BYDV-PAV and PoLV1 in Lolium perenne). The overrepresentation of mixed infection compared to the predicted coinfection rate, as observed in *Poa trivialis*, suggested that the viruses are not circulating independently and some factors such as cotransmission, assistance, or attraction of vectors could have an effect. Results in individual plants, all asymptomatic, confirmed that virus infection is very frequent in nature and generally unapparent (as illustrated and reviewed in references 44, 45, and 46). BYDV-PAV prevalence was higher in uncultivated areas compared to the cropping system, while previous virome studies (20, 27) and ecological hypotheses predicted an increased pathogen prevalence with increase in host abundance (e.g., in cultivated areas) (47, 48). This lower prevalence could reflect successful management of BYDV-PAV in the cereal crops (e.g., insect vector control, planting period). Conversely, pastures and grasslands are mostly composed by perennial grasses that can harbor viruses regardless of vector pressure of a given year. To generalize the trends observed in the Antheit site, the virus prevalence comparison between cultivated and noncultivated communities could be replicated in the other sites (Héron and Latinne) and also on other regions.

Besides its implications from an ecological perspective, the identification of novel plant viruses down to species level also contributes to discussions on the species demarcation criteria proposed by ICTV, in particular for persistent viruses and mycoviruses (*Alphachrysovirus*, *Partitivirus*, *Totivirus*, and *Victorivirus* genera). Indeed, ICTV genome-based species demarcation criteria differ strongly among taxa: from 80 to 90% amino acid sequence identity for partitiviruses to 53 to 70% for alphachrysoviruses, 60% for victoriviruses, and even 50% for totiviruses. Biological criteria such as the host species are also considered (49). In the present study, most of the novel virus species identified presented low identity levels (14 to 80% amino acid identity for RdRp and CP regions) (Table S4) compared to known species in the same genus or family. The partitiviruses were clearly novel viral species, as their amino acid identities were far below the demarcation criteria. However, discriminating among the totiviruses was more complex, as their identity values (14 to 62%) were sometimes close to the demarcation threshold. Before the advent of metagenomics, the ICTV criterion of 50% amino acid identity was sufficient, because the limited number of known species was sufficiently distinct (e.g., only 30% identity between Saccharomyces cerevisiae virus L-A and Saccharomyces cerevisiae virus L-BC in the 717-amino-acid (aa) region of highest similarity) (49). However, the numerous *Totivirus* species identified here and in other metagenomics studies (36, 50) presented less clear tendencies that could eventually suggest reconsideration of demarcation criteria for the genus (e.g., harmonizing rules with victoriviruses that also belong to the *Totiviridae* family and present a demarcation threshold of 60% aa identity).

In summary, this large-scale study revealed a diversified and largely unknown virome in cultivated and noncultivated plant communities, with high viral prevalences and coinfection observed in less-managed communities and with the virome richness increasing with grass species richness in the plant communities. The virome network highlighted different virus lifestyles among wild *Poaceae* communities, illustrating the influence of plant diversity and land use on the composition of viral communities. The present study represents an avenue for future research in virus ecology, in particular, for virus vectors (arthropods, nematodes, fungi, etc.) in virus metagenomics studies to analyze spillover and spill-back between wild and agricultural reservoirs (51). In addition, plant genotyping of different species may provide information on virus adaptation to various hosts (as explored here with LLV). The approach developed here can be extended to more *Poaceae* species from other regions, environmental gradients, and climates and to other plant families (e.g., *Solanaceae*, *Fabaceae*). This could provide a broader overview of the virus communities present in these mixed species communities and aid in understanding the ecology of plant viruses across agro-ecological interfaces, a domain still in its infancy.

## MATERIALS AND METHODS

### Selection of the *Poaceae* communities and sites.

We characterized the virome of three co-occurring *Poaceae*-dominated communities that represent a gradient of plant species richness and management intensity: (i) cereal crops (low plant species richness, intense management); (ii) pastures grazed throughout the year by cattle (moderate species richness, moderate management), and (iii) naturalistic grasslands managed only by annual mowing (high species richness, low management). We studied these community types (Fig. 1) across 2 years (2017 and 2018) at three replicated sites 10 km apart (Antheit, Héron, Latinne) within the Natural Park Burdinale-Mehaigne (province of Liège, Belgium), where the climate is temperate. The natural park landscape includes a patchwork of crop production fields (annual cropping), pastures, high-biological-value meadows, forest, and wild surfaces (including natural reserves), as well as rural villages. The cereal crops included two-row and six-row barley [*Hordeum vulgare* subsp. *vulgare* L. and *Hordeum vulgare* subsp. *hexastichum* (L.) Celak, respectively] and winter wheat (Triticum aestivum L.). The crop fields were cultivated conventionally (with commercial fertilizers and pesticides) at Héron and Latinne and with organic practices (organic fertilization and no pesticides) in Antheit. In contrast, the grazed pastures were sown a century ago and contain four dominant grass species, all of which are cool-season C3 perennials native to Eurasia: *Agrostis capillaris* L., *Dactylis glomerata* L., Lolium perenne L., and *Poa trivialis* L. Lastly, the grasslands were communities with high conservation value, containing up to 14 *Poaceae* species and additional forbs. These grasslands had been mowed once a year in mid-July for the past 15 to 30 years (see Table S1), except in Héron, which was mowed in mid-May in 2017.

### Sampling strategy.

To capture the virome of these cool-season *Poaceae* communities, we surveyed them twice: from mid-May to mid-June of 2017 and 2018 (18 surveys; 3 community types × 3 replicates × 2 years). In 2018, we sampled an additional six-row barley field (AF-3) adjacent to the pasture plot in Antheit, because it presented interesting symptoms of fungal infection. Thus, there were 19 surveys in total. For each survey, we randomly placed 50 quadrats (1 m by 1 m) in the target area (1.1 to 2.7 ha). Within each quadrat, we inventoried all *Poaceae* species and noted any viral or fungal symptoms. We harvested one randomly chosen individual (one stem with leaves) of each species identified within that specific quadrat. Harvested plant material was kept cool on ice freeze packs in the field and then stored at −80°C later that day.

To compare the virome captured in each survey, we prepared 19 corresponding pools of 50 individual plants each, based on botanical inventory of the 50 quadrats visited in each survey (Table S2). Each pool reflected the relative abundance of the *Poaceae* species encountered in that survey. For instance, 20% of the grasses within the 2017 Antheit Grassland survey were *L. perenne* (47/232 plants sampled) (Table S1), so this species was represented in the 50-sample pool by 10 plants. Non-*Poaceae* species were not surveyed.

### Purification of virus-like particles and nucleic acid extraction.

Both DNA and RNA viruses were targeted in this study. The 19 pools of plants were prepared by virus-like particle (VLP) enrichment, followed by the extraction and sequencing of VANA, as described previously (32). To obtain the VLPs, 200 mg of frozen tissues from each individual plant was pooled into a filtered bag (i.e., 10 g for 50 plants) and then ground in 70 mL of Hanks’ buffered salt solution (HBSS, composed of 0.137 M NaCl, 5.4 mM KCl, 0.25 mM Na_2_HPO_4_, 0.07 g glucose, 0.44 mM KH_2_PO_4_, 1.3 mM CaCl_2_, 1.0 mM MgSO_4_, 4.2 mM NaHCO_3_), using a tissue homogenizer. The homogenized plant extracts were centrifuged at 3,200 × *g* for 5 min. Supernatants were further centrifuged at 8,200 × *g* for 3 min. Resulting supernatants were then filtered through a 0.45-μm sterile syringe filter, and 25 mL of supernatant was put into an ultracentrifuge tube. Then, a sucrose cushion, made of 3 mL of 30% sucrose in 0.2 M potassium phosphate (pH 7.0), was added. Extracts were then centrifuged at 148,000 × *g* for 2.5 h at 4°C using an SW 28 Ti rotor (Beckman). The resulting pellet was resuspended overnight at 4°C in 1.5 mL of HBSS. Unencapsidated nucleic acids were eliminated by adding 15 U of bovine pancreas DNase I (Euromedex) and 1.9 U of bovine pancreas RNase A (Euromedex, France) to 200 μL of resuspension. Samples were incubated at 37°C for 90 min. VANA were then extracted from 200 μL of resuspended virions by using a PureLink viral RNA/DNA minikit (ThermoFisher Scientific, Belgium) following the manufacturer’s protocol.

### Library preparation and high-throughput sequencing.

Reverse transcription, Klenow fragment treatment, and amplification of the viral DNA and RNA using barcoded PCR primers were performed according to a protocol described elsewhere (52). Amplification products were cleaned up using the Nucleospin gel and PCR cleanup system (Macherey-Nagel, Germany). Ten libraries with unique multiplex identifier linkers (MIDs, described elsewhere [53]) were further assembled and sequenced at GIGA-Liège University (Belgium) using the NEBNext Ultra II DNA library prep kit (New England BioLabs) and then sequenced on the Illumina NextSeq 500 platform with 2 × 150-nt reads with a target of 10 million sequences per pool of 10 libraries (i.e., 1 million sequences per 50-plant pool).

To control proper extraction of RNA and DNA viruses (and to deal with cross-sample contamination), a series of blank (water) and alien controls were used in each batch of sequenced samples. An alien control, which has been recommended in recent guidelines (54), corresponds to a plant sample infected by a virus (called alien virus) that cannot infect the studied plant samples. For this study, potato leaf roll virus (genus *Polerovirus*, RNA virus) and banana streak virus (genus *Badnavirus*, DNA virus) were selected. The presence of reads from alien viruses in the samples correspond to cross-contamination between samples and can support the establishment of a threshold below which presence of a virus is considered background cross-contamination.

### Sequence analyses and annotation.

After quality check, raw reads were demultiplexed and cleaned according to their MID linkers with Cutadapt (55). Then, reads were *de novo* assembled into contigs (SPAdes assembler; 2.5% mismatches allowed, minimum size of 35 nt) (56) on the Durandal cluster (ULiège, Belgium). Contigs were annotated using BLASTn and BLASTx (57) and NCBI nucleotide (nt) and protein (nr) databases, respectively. Viral BLASTx hits (conservative e-value of <e^−20^ cutoff) were isolated for further analyses using Geneious Prime 2019.2.1. In order to obtain complete or nearly complete virus genomes, we assembled the virus contigs and then mapped the reads on these contigs (Geneious mapper, 5 iterations, medium-low sensitivity, 5% mismatches and 5% gaps allowed). Novel virus species were further tentatively identified following host and genomic ICTV species demarcation criteria for each virus family (host range and nucleotide or protein identity percentage on RdRp and coat protein). We putatively attributed the host (plants or fungi) of the novel viruses according to the known host range of the related virus family or genus and the closest virus hit after the BLAST analysis. In order to obtain robust results, we performed the analyses (virome richness, network, phylogeny) on complete (or nearly complete) virus genomes, excluding the small contigs (less than 500 nt) that did not allow formation of complete virus sequences.

### Viral genetic structure analysis.

We examined the population genetic structure of *Lolium* latent virus (LLV) over years, communities, and sites. The abundance of this virus in almost all the pools, including *Lolium* samples (e.g., up to 80% of total reads in Antheit grassland in year 2) allowed us to analyze the LLV intraspecific genetic structure, phylogeny, and variants among and within plant communities. As the complete genome of LLV was not obtained for all samples, the analysis focused on the RdRp gene, which was better covered (corresponding to nt 88 to 5277 in the NCBI reference genome, NC_010434). After obtaining *de novo* LLV contigs for each sample, a multiple nucleotide alignment of the 31 contigs was performed with MUSCLE (58, 59) with 8 iterations, and a phylogenetic tree was built on MEGA-11 software (maximum-likelihood tree, Tamura-Nei model, 1,000 bootstraps, and a support threshold of 70% occurrence in the consensus tree).

### Statistical and network analyses.

Viromes at the family, genus, and species levels were inventoried from the 19 pools. Statistical analyses were performed using Minitab19 to determine whether virome richness levels (i.e., number of virus taxa identified in each plant community) were significantly different between plant communities and anthropogenic management methods. Population structure was analyzed through the distribution normality and variance homogeneity. As the two hypotheses were not fulfilled for all the conditions, we used the Kruskal-Wallis test to analyze all communities and the Mann-Whitney test to compare two community categories (e.g., pastures and grasslands). Both tests were performed with a 95% confidence level. In addition, network analyses of the virome composition (i.e., the different virus taxa constituting the virome) in the different *Poaceae* communities were implemented on ORA-LITE software (Netanomics). The aim of this analysis was to visualize how virus species were distributed among the communities and to determine any association between virus taxa and plants, e.g., comparing persistent and nonpersistent (acute) virus lifestyles, according to definitions reported elsewhere (60) (see Table S7 for the virus-community matrix used). The same matrix was used to perform hierarchical clustering analyses with Morpheus software (Pearson correlation analysis, default parameters) (https://software.broadinstitute.org/morpheus), recursively merging objects (i.e., *Poaceae* communities and virus species) based on their pairwise distance.

### Virus prevalence, coinfection, and spatial distribution in individual plants.

Total RNA was extracted according to methods described previously (61). Reverse transcription was carried out with Tetro reverse transcriptase (RT) enzyme (Bioline). The 20-μL RT reaction mix consisted of 2.5 μL of total RNA (at 300 ng/μL), 10 μL of RNase-free water, 4 μL of 5× RT buffer, 1 μL of random hexamers (50 μM), 1 μL of deoxynucleoside triphosphate (dNTP) mix (10 mM), 0.5 μL of RNase Out (40 U/μL), and 1 μL of Tetro enzyme (200U/μL). The reaction mixtures were then incubated as follows: 25°C for 10 min, 45°C for 30 min, 85°C for 5 min, and then placed directly on ice. Amplification of the cDNA was performed using a Mango *Taq* enzyme kit (Bioline) in a 25-μL reaction mix composed of 2.5 μL of cDNA, 12.5 μL of RNase-free water, 5 μL of 5× PCR buffer, 0.5 μL of dNTP mix (10 mM), 1 μL of each primer (20 μM), and 0.5 μL of Mango *Taq* enzyme (5 U/μL). Primers (listed in Table S8) were previously designed for each targeted virus (BYDV-PAV, novel PoLNVA, and novel PoLV1) from the HTS data using Geneious Prime 2019.2.1 software. Thermal cycling corresponded to 94°C for 4 min, 35 cycles at 94°C for 45 s, Ta for 1 min, and 72°C for 30 s, and a final at 72°C extension for 10 min. PCR products were analyzed by electrophoresis on a 1% agarose gel in Tris-acetate-EDTA buffer, stained with GelRed nucleic acid gel stain (Biotium), and visualized under UV light.

### SADIE.

The spatial distributions of BYDV, PoLNVA, and PoLV1 in *L. perenne* and *P. trivialis* were analyzed with the SADIE technique using SADIEShell version 2.0 and N_AShell version 1.0 (Rothamsted Experimental Station, Harpenden Herts, United Kingdom). This approach is used to study spatial patterns in count-based data where location is known and to test the statistical significance (62). Comparing observed patterns of samples with two extremes (crowding and regularity), the *I_a_* was calculated to quantify the presence and degree of clustering. *I_a_* values of 1 indicate a random spatial distribution, an *I_a_* of >1 reveals aggregation of counts into clusters, and an *I_a_* of <1 points out a regular or uniform spatial distribution (63, 64). Statistical significance of aggregation was evaluated by the null hypothesis (*P_a_*) that the counts are arranged randomly with respect to one another (significant if *P_a_* is <0.05 or *P_a_* is >0.95, meaning an aggregative or regular pattern, respectively) (65). Clustering indices can be used to determine an association index X in order to compare different viral populations within same location and evaluate if they occur close together in similar habitats (plant community × plant species), or conversely if they are segregated from one another. This analysis is associated with a significance test under the null hypothesis of no association (with *P* < 0.025 or *P* > 0.975 for a significant positive or negative association, respectively) (66). For BYDV-PAV and PoLV1, we hypothesized aggregation spots related to insect presence in the field.

### Data availability.

Raw sequencing reads were submitted in the NCBI Sequence Read Archive, with the BioProject accession number PRJNA882095. All data related to virus genome assembly and identification, and their NCBI GenBank accession numbers (OL330737 to OL330775 and ON137708 to ON137726) are listed in Table S4. All other data are provided in the supplemental material.
