# Supplementary material for: Long-Term Anthropogenic Management and Associated Loss of Plant Diversity Deeply Impact Virome Richness and Composition of Poaceae Communities
Source: Microbiol Spectr. 2023 Mar 14;11(2):e04850-22. doi: 10.1128/spectrum.04850-22 (PMC10100685; doi:10.1128/spectrum.04850-22)

Supplementary Figure S1. Hierarchical clustering analysis in both plant and virus dimensions. Columns referred to the different Poaceae communities (Fields (F), Pastures (P) and Grasslands (G)) examined among sites (Antheit (A), Héron (H) and Latinne (L)). Rows corresponded to the different virus species detected.

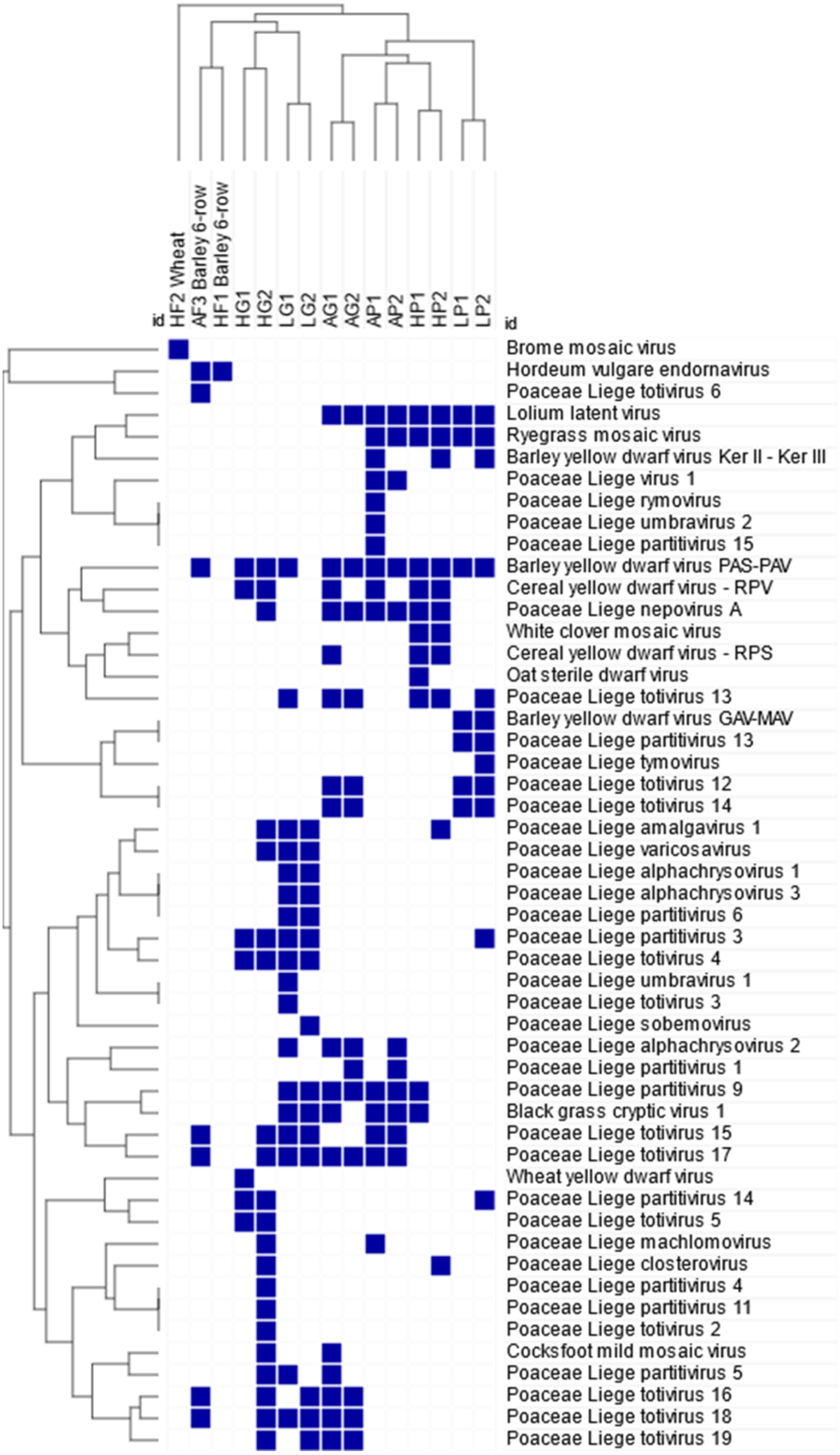

Supplement: Supplemental file 9 — Fig. S1. Download spectrum.04850-22-s0001.pdf, PDF file, 0.3 MB [file spectrum.04850-22-s0001.pdf]
