# Supplementary material for: Long-Term Anthropogenic Management and Associated Loss of Plant Diversity Deeply Impact Virome Richness and Composition of Poaceae Communities
Source: Microbiol Spectr. 2023 Mar 14;11(2):e04850-22. doi: 10.1128/spectrum.04850-22 (PMC10100685; doi:10.1128/spectrum.04850-22)
Supplement: Supplemental file 8 — Table S8. Download spectrum.04850-22-s0009.pdf, PDF file, 0.04 MB [file spectrum.04850-22-s0009.pdf]

Supplementary Table S8: Primers used for targeted virus detection by RT-PCR

Primers were previously designed for each targeted virus (BYDV-PAV, novel PoLNVA and novel PoLV1) from the HTS data using Geneious Prime 2019.2.1 Software (https://www.geneious.com). They are listed with their respective annealing temperatures (Ta) in the table hereunder.

**Primers**	**Sequence (5'-3')**	**5' Position**	**Ta (°C)**	**Amplicon size**
**BYDV-F**<br>**BYDV-R**	CCCAGTCTATCGCAATGCCCAGC<br>GGTTCCGGTGTTGAGGAGTCTAC	3104<br>3483	55°C	379 bp
**PoLNVA-F**<br>**PoLNVA-R**	ACCCTCAAGTTCTTTCCACTT<br>ACTCCCTCTCCAGTATTGAA	3775<br>4150	63°C	375 bp
**PoLV1-F**<br>**PoLV1-R**	TGTGTCGGGAAATAAACTACAAGCA<br>GCAAAAGAGCCAAACTGGAATGGTA	3251<br>3607	56°C	356 bp
